# 

**Published:** 1876-10

**Authors:** 


### TABLE OF CONTENTS.

ORIGINAL COMMUNICATIONS—

Skimble-Scamble "Dots" by the Wayside. By Thin Pate, M. D., of Berea, N. C. .... 577

Placenta Previa. By Wm A Barry, M. D., Hot Springs, Ark .... 579

Paraphimosis. By J C Watson, M. D., Saltville, Va .... 581

SELECTIONS—

Diagnostic Syllabus of the Inflammation Commonly Met with in the Uterus and Vagina. Prepared by J. H. Etheridge, M. D .... 582

Cholera Infantum—Treatment. By B. D. Keator, M. D .... 585

Phosphide of Zinc .... 590

Notes on the Local Treatment of Certain Diseases of the Skin. By L. Duncan Bulkley, A. M., M. D .... 593

Eucalyptus in Dropsies. By J. B. Leary, M. D .... 597

The Management of Albuminuria .... 600

The Use of Aromatic Sulphuric Acid in Necrosis. By Ephraim Cutter, M. D., Cambridge .... 601

Can "Port-Wine Marks" on the Face be Cured?—Yes. By Balmanno Squire, M. D., Surgeon British Hospital, London .... 603

Iodoform with Iron as an Alterative. By A. L. Reichard, M. D., Allentown, Pa. 605

EXTRACTS AND GLEANINGS—

Iodoform as a Topical Application, Particularly in Venereal Diseases—609. To Prevent the Secretion of Milk in the Female Breast—610. Typhoid Fever—611. Permanganate of Potash in Leucorrhœa—612. Ammoniated Tincture of Guiacum in Inflammation of the Throat—613. Therapeutics of Carbolic Acid—614. Croton Chloral Hydrate in Photophobia—616. Treatment of Chronic Constipation—617. Treatment of Excoriations of the Os Uteri—618. Bathing in Entero-colitis—619. Treatment of Eclampsia—621. Antiseptic Treatment of Diphtheria—622. Tannin Dressing for Wounds—623. Treatment of Disease of the Stomach by Washing out by Means of the Stomach Pump—623. Muriate of Ammonia in Diphtheria—624. Choice of Sedatives for the Aged—624. The Treatment of Diphtheria—625. Carbolic Acid in Carbuncles—626. Local Treatment of Burns—627. Fibroid Tumor of the Prostrate Successfully Treated by Injection of Iodine—628. Treatment of Cystitis by Atropia in Enemata—629. Absorption of Iodine by the Skin—630. Ulcerated Nipples—630. Advances in Gynæcology During the Year 1875—631. Raw Onion as a Diuretic—631. Chlorate of Potash in Infantile Diarrhœa—632. Citrate of Quinoidine—632. Chloroform in the Surgery of Infants—633. Traumatic Tetauus in the Horse—633. How to Make Leeches Bite—633. Prophylactic in Enlarged Tonsils—633. Conium—634. Glycerol of Subacetate of Lead—635. The Penitentiary for a Vendor of Adulterated Milk—634 Treatment of Diphtheria—635. Treatment of Nasal Catarrh by Nitrate of Bismuth—636. Phosphorus Pills—635. Pancreatic Meat Emulsion—636. Formula for Diphtheria—636. The Preservation of Raw Meat—636.

EDITORIAL AND MISCELLANEOUS—

To Our Subscribers .... 637

Book Notices .... 637

